# Combination immunohistochemistry for CK5/6, p63, GATA6, and HNF4a predicts clinical outcome in treatment-naïve pancreatic ductal adenocarcinoma

**DOI:** 10.1038/s41598-024-65900-w

**Published:** 2024-07-06

**Authors:** Takahiro Shibayama, Akimasa Hayashi, Masao Toki, Keiichiro Kitahama, Yu-Jui Ho, Kenichiro Kato, Takahiro Yamada, Sho Kawamoto, Komei Kambayashi, Kazushige Ochiai, Koichi Gondo, Naohiro Okano, Jerry P. Melchor, Christine A. Iacobuzio-Donahue, Yoshihiro Sakamoto, Tadakazu Hisamatsu, Junji Shibahara

**Affiliations:** 1 Department of Pathology, Kyorin University School of Medicine, 6-20-2 Shinkawa, Mitaka, Tokyo, 181-8611 Japan; 2 Department of Gastroenterology and Hepatology, Kyorin University School of Medicine, Tokyo, Japan; 3 Cancer Biology and Genetics Program, Sloan Kettering Institute, Memorial Sloan Kettering Cancer Center, New York, NY USA; 4 Department of Medical Oncology, Faculty of Medicine, Kyorin University, Tokyo, Japan; 5 The David M. Rubenstein Center for Pancreatic Cancer Research, Sloan Kettering Institute, Memorial Sloan Kettering Cancer Center, New York, NY USA; 6 Department of Surgery, Kyorin University School of Medicine, Tokyo, Japan

**Keywords:** Pancreatic ductal adenocarcinoma, Expressional subtypes, Surrogate biomarker, Immunohistochemistry, Oncology, Cancer, Gastrointestinal cancer, Pancreatic cancer

## Abstract

Although sequence-based studies show that basal-like features lead to worse prognosis and chemotherapy-resistance compared to the classical subtype in advanced pancreatic ductal adenocarcinoma (PDAC), a surrogate biomarker distinguishing between these subtypes in routine diagnostic practice remains to be identified. We aimed to evaluate the utility of immunohistochemistry (IHC) expression subtypes generated by unsupervised hierarchical clustering based on staining scores of four markers (CK5/6, p63, GATA6, HNF4a) applied to endoscopic ultrasound-guided fine needle aspiration biopsy (EUS-FNAB) materials. EUS-FNAB materials taken from 190 treatment-naïve advanced PDAC patients were analyzed, and **t**hree IHC patterns were established (Classical, Transitional, and Basal-like pattern). Basal-like pattern (high co-expression of CK5/6 and p63 with low expression of GATA6 and HNF4a) was significantly associated with squamous differentiation histology (*p* < 0.001) and demonstrated the worst overall survival among our cohort (*p* = 0.004). IHC expression subtype (Transitional, Basal vs Classical) was an independent poor prognosticator in multivariate analysis [HR 1.58 (95% CI 1.01–2.38), *p* = 0.047]. Furthermore, CK5/6 expression was an independent poor prognostic factor in histological glandular type PDAC [HR 2.82 (95% CI 1.31–6.08), *p* = 0.008]. Our results suggest that IHC expression patterns successfully predict molecular features indicative of the Basal-like subgroup in advanced PDAC. These results provide the basis for appropriate stratification for therapeutic selection and prognostic estimation of advanced PDAC in a simplified manner.

## Introduction

Pancreatic ductal adenocarcinoma (PDAC) is a fatal malignancy with an overall 5-year survival rate less than 10%^1,2^. Most patients (80–90%) have surgically unresectable advanced-stage carcinomas at the time of diagnosis^1^, and combination chemotherapy including gemcitabine or fluorouracil results in median survival of less than 1 year in patients with advanced PDAC^3,4^.

For therapeutic and prognostic stratification, several transcriptomic subtypes based on gene expression profiling using resected PDAC tissue samples have been proposed to date^5–7^, and it is suggested that PDAC is chiefly classified into two distinct subgroups termed classical/progenitor or basal-like/squamous (poorer prognosis)^8^.

Recent studies demonstrated that basal-like signature is a poor prognosticator even in advanced PDAC and associated with resistance to chemotherapy^9,10^ as well as its single cell level heterogeneity^11^ and/or plasticity during clonal evolution^12^. In routine diagnostic practice, however, the implementation of these approaches, which require next-generation sequencers and/or other high-technology devices^13^, can be challenging. In addition, given that PDAC cases are often discovered in inoperable stage^1^ and the technical challenges of endoscopic ultrasound-guided fine needle aspiration biopsy (EUS-FNAB)^14^, only limited biopsy samples are available. Considering these, immunohistochemistry (IHC)-based surrogate biomarkers emerge as highly useful tools for clinicians and pathologists. Numerous studies and reviews, including The Cancer Genome Atlas (TCGA) study, have identified potential surrogate markers^5,8,10,12,15–19^. Among these markers, we selected those supported by multiple studies and evaluated by the Human Protein Atlas (https://www.proteinatlas.org/). For example, GATA 6, which was identified as a candidate surrogate marker in TCGA study^8^, has been analyzed for its expression levels using RNA sequencing (RNA-seq), in situ hybridization (ISH), and IHC with a computer-assisted method^10,13,15^. These approaches have been introduced as valuable tools to differentiate between basal-like and classical PDAC using GATA6 expression as a potential surrogate biomarker. HNF4a and p63 are also mentioned as candidate surrogate markers in the International Cancer Genome Consortium (ICGC study)^5^ and further supported by mouse model and/or human IHC studies^16–19^. While these transcription factors are the potential key molecules that can distinguish expressional subtypes, recent IHC based human studies have shown the CK5/6 (KRT5 and KRT6A) is one of the promising surrogate markers for basal-like^7^ and/or squamous^5^ subtypes^12,15^. Considering the commercial availability of antibodies for IHC and the Human Protein Atlas evaluation, we believe that GATA6, HNF4, CK5/6, and p63 are the most suitable IHC surrogate biomarkers. In this study, we assessed the usefulness of combining these four IHC markers in evaluating treatment-naïve advanced PDAC using EUS-FNAB samples. Our aim was to determine whether these markers could serve as viable surrogate biomarkers for predicting molecular signatures. Additionally, we evaluated the publicly available expression and single-cell data to further validate their utility.

## Materials and methods

### Ethics approval and consent to participate

This study was conducted with approval from the Kyorin University Institutional Review Board (approval code: R03-162). All procedures involving human participants were performed in accordance with the ethical standards of the institutional research committee and with the 1964 Helsinki declaration and its later amendments or comparable ethical standards. Informed consent was secured through an opt-out process. All participant data were anonymized and de-identified to protect privacy.

### ICGC data download and analysis

Clinical information and microarray data in ICGC study were downloaded from Supplementary Tables 1 and 14 from the original manuscript^5^. The downloaded microarray data were used to retrieve a normalized count matrix, which was then processed to obtain counts per million (c.p.m) and log2 transformed using RSEM. This count matrix was further Z-score normalized for calculating the sum as the gene signature scores and used to generate a heat map. Gene signatures corresponding to the Squamous/Progenitor (Bailey)^5^ and Basal-like/Classical subtypes (Moffitt)^7^ were used to stratify patients into groups characterized by high or low expression levels. Differential expression profiles of all transcription factors were quantified between groups. Gene expression data from Bailey et al. were reanalyzed and organized into hierarchical clusters based on established gene signatures^5^ and specific markers identified in this study (TP63, KRT5, KRT6A, HNF4A, and GATA6). Our custom 5 gene signature score is defined as sum (cpm.z(TP63, KRT5, KRT6A))—sum (cpm.z(GATA6, HNF4A)). Patient samples are assigned to high or low groups based on individual signature scores, and differences between groups are shown using log2FoldChange in expression and patient survival outcome. The FANTOM5 database was used to identify transcription factors.

### Single cell data download and analysis

Single-cell RNA-seq data were downloaded from EGA under accession code EGAD00010001811. Analysis was done following methods described in the original manuscript^11^. Marker expression and gene signatures were displayed using Seurat’s AddModuleScore functionality. Similar results were reproduced for patient 100,070 and 85,948 individually.

### Case selection and histological review

After approval by Kyorin University Institutional Review Board (R03-162), the consecutive PDAC cases diagnosed by FNAB samples taken from primary lesions were retrieved from the pathology archives of Kyorin University Hospital (Tokyo). Patients whose FNAB specimens contained sufficient tumor cells for histological diagnosis were included. Intraductal papillary mucinous neoplasm (IPMN)-derived carcinoma was excluded from the cohort. Clinicopathological information including age, sex, clinical stage, treatment and outcome was retrieved from the medical records.

The histology (hematoxylin and eosin slides) and IHC scoring were reviewed by two gastrointestinal pathologists (TS and AH) who were independently blinded to molecular and clinical data. Although the evaluations of most cases were concordant, discrepant diagnostic results were unified into one category through discussion. Because EUS-FNA is not routinely performed for cystic pancreatic lesions in Japan, fragmented atypical epithelium without a definite stromal invasion is diagnosed as a carcinoma by pathologists. Tumor histology was subclassified into glandular pattern, glandular pattern with poorly differentiated component (Por comp), or glandular pattern with squamous differentiation (Sq diff). Glandular pattern was defined as ductal formation and/or fragmented tumor cells with intracytoplasmic mucin including cytoplasmic foamy cell change. Squamous differentiation was evaluated as dense, opaque eosinophilic polygonal tumor cells regardless of whether keratinization (keratohyalin granules and/or pearls) and/or intercellular bridges were observed. Poorly differentiated component was defined as tumor cell cluster harboring neither glandular pattern nor squamous differentiation. If only a small amount of tumor cells with squamous differentiation was present, it was classified as Sq diff. The same criteria were applied to Por comp.

### Immunohistochemistry

IHC was performed on 4-μm-thick formalin-fixed and paraffin-embedded (FFPE) whole tissue sections. The primary antibodies used were CK5/6 (D5/16 B4, 1:50; DAKO, Glostrup, Denmark), p63 (4A4, 1:100; Zeta Corporation, Arcadia, CA, USA), GATA6 (D61E4, 1:100; Cell Signaling Technology, Danvers, MA, USA) and HNF4a (H1415, 1:1000; Perseus Proteomics, Tokyo, Japan). Heat-induced epitope retrieval was performed and the Envision system (DAKO) was used for detection. Cytoplasmic and/or membranous staining was evaluated as positive for CK5/6, and nuclear staining was regarded as positive for p63, GATA6 and HNF4a. IHC slides were evaluated semiquantitatively based on the Allread scoring system which is clinically used in hormone receptor evaluation of breast cancer^20^. Staining intensity score (0: negative, 1: weak, 2: moderate, 3: strong) and proportional score based on percentages of stained cells (0: 0%, 1: < 1%, 2: 1–10%, 3: 10–33%, 4: 33–67%, 5: > 67%) were evaluated independently and total score (range 0–8) was calculated as sum of these scores. In this study we subclassified total scores into 4 grades as score 0 (total score 0–2), score 1+ (total score 3–5), score 2+ (total score 6, 7), score 3+ (total score 8).

### Statistical analysis

All data analysis was performed using JMP (version 17.0.0) and/or R (version 4.2.2). The difference of categorical variables was evaluated using the Fisher’s exact test. Overall survival rate, measured from the date on which FNAB specimen was obtained, was calculated using Kaplan–Meier Method, and survival differences were compared using log-rank test. The Cox proportional hazards regression method was used for multivariate analysis. All tests were two-sided. Results with *p* value < 0.05 were considered statistically significant.

### Ethics declarations

This study protocol was approved by the Faculty of Medicine Research Ethics Committee (R03-162), Kyorin University, with waiver of informed consent.

## Results

### Transcription factor enrichment and representative markers in PDAC subtypes

**Figure 1:**
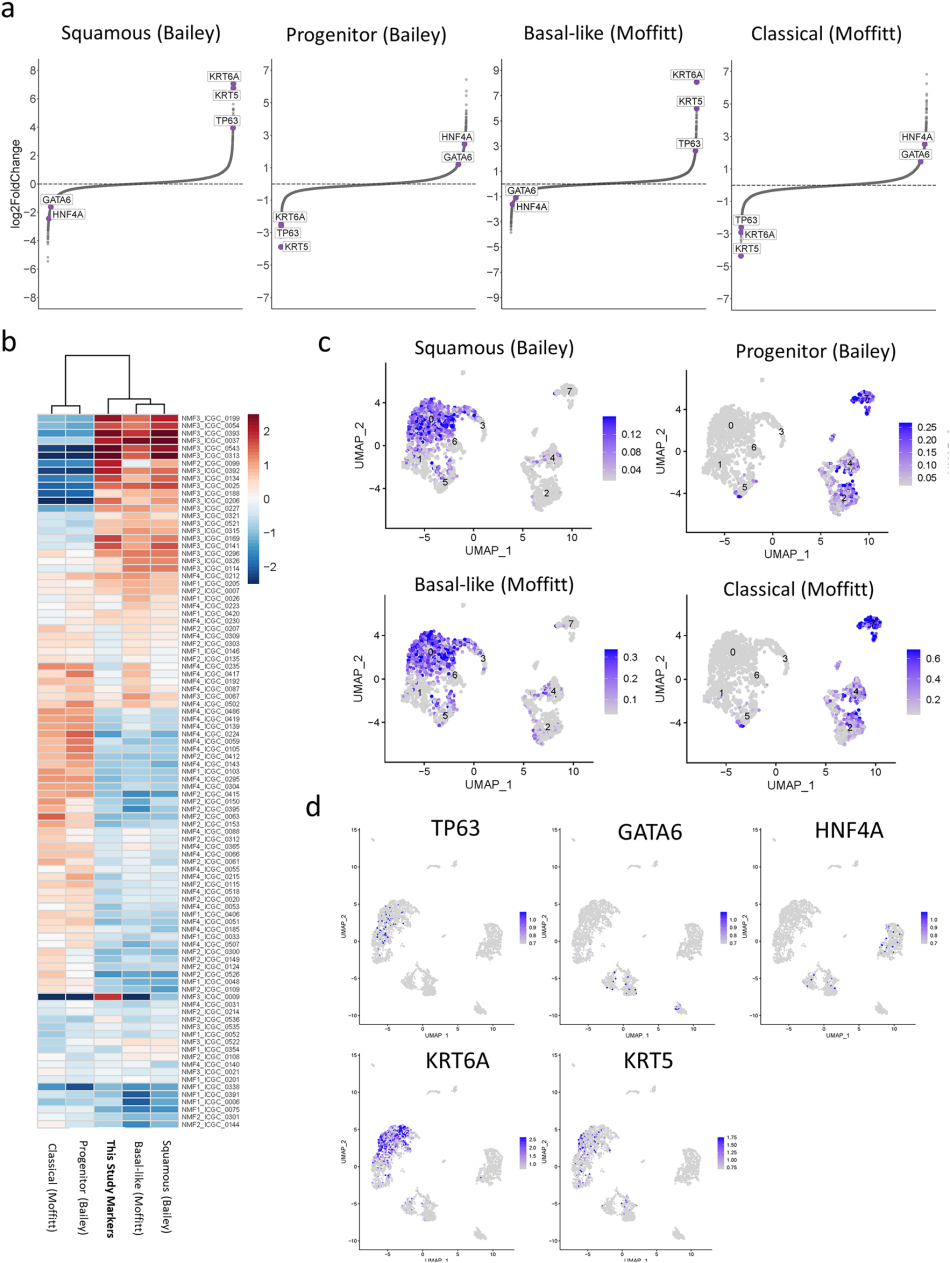
Subtypes in PDAC and representative markers. (**a**) Transcription factor expression in PDAC patients, where patient samples were dichotomized based on gene signature scores defining Squamous/Progenitor (Bailey) or Basal/Classical (Moffitt) subtypes. Scatterplots show expressed transcription factors ranked by their mean log2 fold change between patient samples from Bailey et al.^5^. The five markers identified in this study (TP63, KRT5, KRT6A, HNF4a, and GATA6) are highlighted in purple. (**b**) Heatmap representation of unsupervised hierarchical clustering of gene signature scores from Bailey et al.^5^. Signature scores were calculated for each patient sample using subtype markers (Moffitt and Bailey) and five markers in this study. UMAP plot of gene signature scores based on single-cell RNA-seq data for patient 100,070 from Chan-Seng-Yue et al.^11^ using (**c**) markers defining Squamous, Progenitor, Classical, and Basal-like subtypes (Bailey and Moffitt) and (**d**) our five markers. The heatmap in Fig. 1b was generated using tidyverse (https://www.tidyverse.org/) and pheatmap (version 1.0.12) in the R environment.

Gene signatures for Squamous/Progenitor subtypes (Bailey)^5^ or Basal-like/Classical (Moffitt)^7^ subtypes were used to assign patients into high or low groups. Differences in the expression profiles between the two groups were calculated for all transcription factors and representative keratin markers (KRT5 and KRT6A), as shown in Fig. 1a. TP63, KRT5, and KRT6A were enriched in the Squamous and Basal-like phenotypes, whereas HNF4A and GATA6 were enriched in the Progenitor and Classical phenotypes. Gene expression data from Bailey et al. were reanalyzed and hierarchically clustered based on published gene signatures^5^ and markers identified in this study (TP63, KRT5, KRT6A, HNF4A, and GATA6). Our five representative markers distinguished the Squamous/Progenitor (Bailey) and Basal-like/Classical (Moffitt) subtypes almost to the level of their original criteria, as shown in Fig. 1b. In addition, these markers also reflected the clinical course, as shown in Supplementary Fig. 1.

### Single-cell level tumor heterogeneity based on subtypes and subtyping markers

Single-cell data were reanalyzed to demonstrate the distribution of marker genes and known gene signatures (Patient 100,070)^11^ as shown in Fig. 1c. Squamous/Basal-like type tumor cells and Progenitor/Classical type tumor cells co-existed within the same PDAC patient. TP63 and KRT5/6A positive cells were enriched in Squamous/Basal-like populations, while HNF4a and GATA6 positive cells were enriched in Progenitor/Classical populations (Fig. 1c,d and Supplementary Fig. 2).

### Clinicopathological features of the cohort

**Figure 2:**
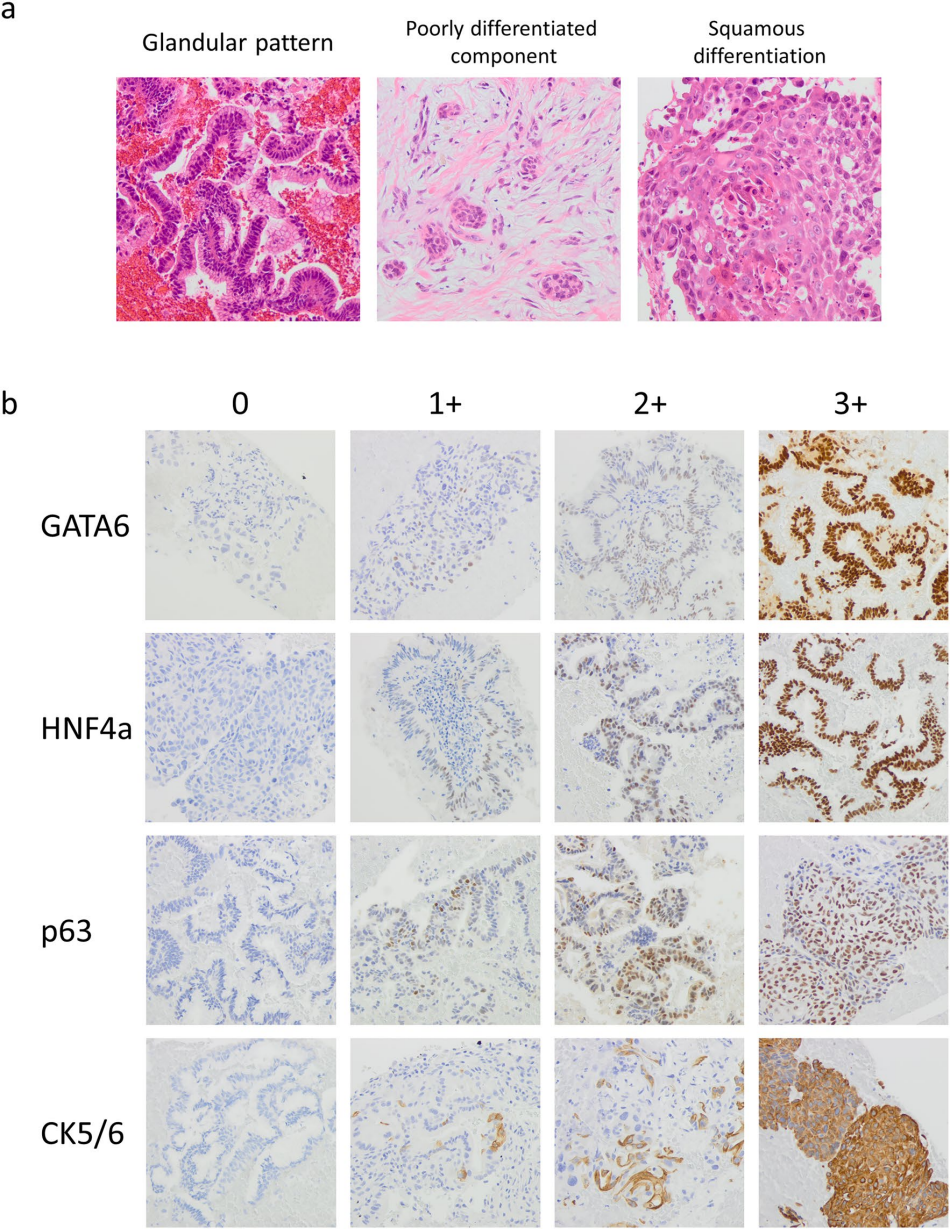
Histological subtype of PDAC. (**a**) Glandular pattern is characterized by pancreato-biliary type epithelium with abundant intracytoplasmic mucin. Poorly differentiated component (Por comp) is defined as tumor cell cluster or nest with neither glandular nor squamous differentiation. Squamous differentiation (Sq diff) is characterized by tumor cells showing opaque eosinophilic cytoplasm. (**b**) IHC score of each four markers (GATA6, HNF4a, p63, CK5/6). Total score (intensity + proportional score) is graded as score 0 (total score 0–2), score 1+ (total score 3–5), score 2+ (total score 6,7), score 3+ (total score 8) according to Allred scoring system.

A total of 191 treatment naïve cases were enrolled. A patient whose FNAB material was insufficient for additional IHC analysis was excluded. A total of 190 cases were analyzed. The clinicopathologic characteristics of this study cohort are shown in Supplementary Table 1. The patients included 96 males and 94 females with a median age of 71 years (range, 40 to 87 y). 144 patients (76%) of this cohort were diagnosed with advanced stage PDAC (stage III, IV). All patients were treated (surgical resection and/or chemotherapy) after FNAB performed from primary lesions. Median follow-up was 292 days (range, 7 to 42,901). Histologically, 134 patients (71%) showed Glandular pattern, whereas 40 patients (19%) and 16 patients (10%) met the criteria of poorly differentiated component and squamous differentiation, respectively (Fig. 2a).

### Histological characteristics of IHC expression types

**Figure 3:**
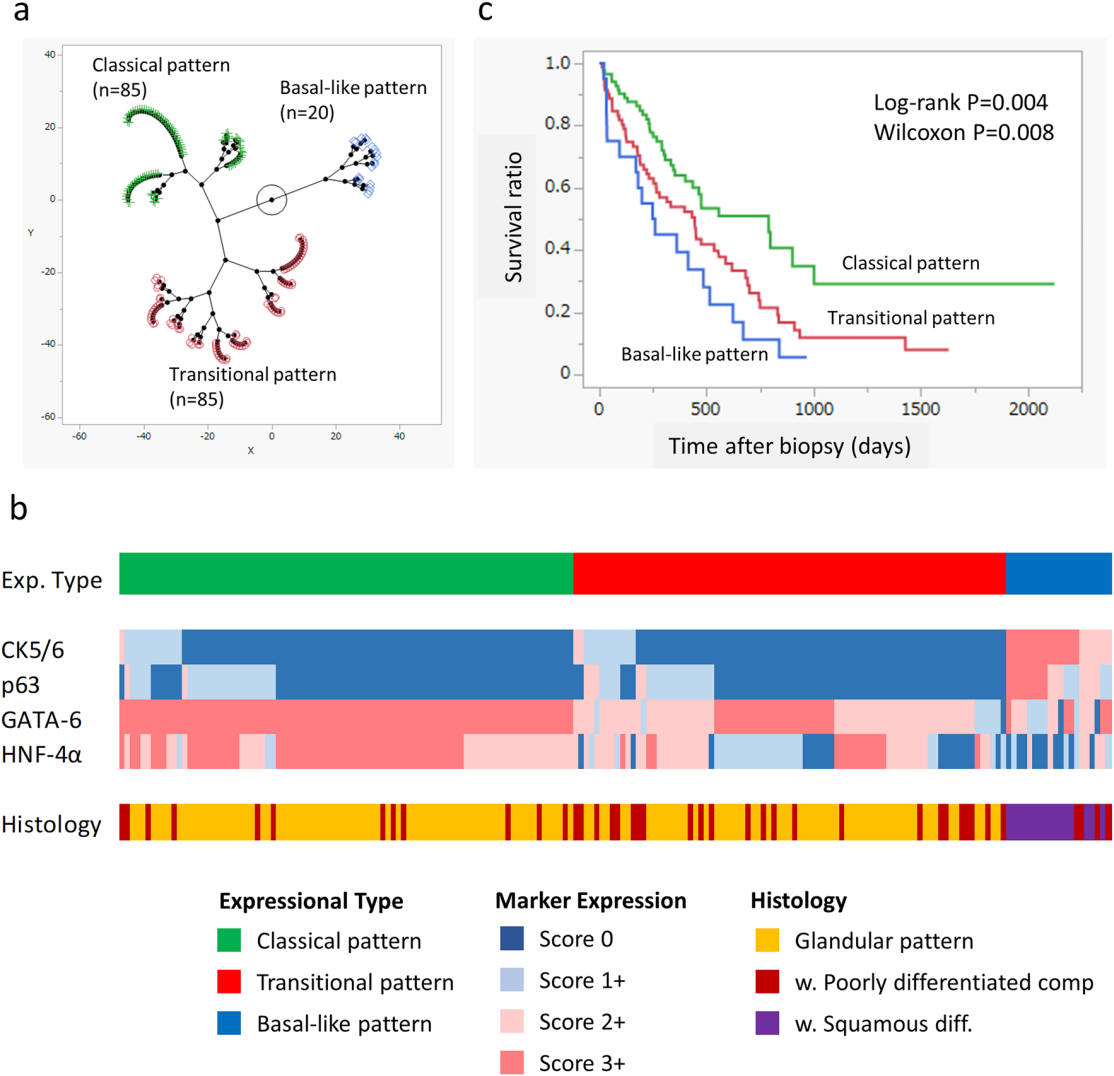
IHC expression pattern with clinicopathological features. (**a**) By unsupervised hierarchical clustering analysis based on IHC scores of four markers, PDAC was segregated into three distinct subgroups, namely Classical pattern (N = 85), Transitional pattern (N = 85) and Basal-like pattern (N = 20). (**b**) Heatmap of differentially expressed IHC markers and histological patterns between IHC based subgroups. Basal-like pattern generally corresponds to high expression of CK5/6 and p63, low expression of HNF4a and GATA6, and Sq diff histology. The heatmap in Fig. 3b was generated using Excel 2019, based on the clustering analysis performed with JMP (version 17.1.0). (**c**) Kaplan–Meier analysis revealed significantly worst prognosis of Basal-like pattern amongst all three groups (Log-rank *p* = 0.004, Wilcoxon *p* = 0.008).

The results of IHC scoring for each of the four markers were summarized in Table 1, and the representative images were demonstrated in Fig. 2b. Unsupervised hierarchical clustering analysis was applied to IHC expression scoring data sets of each four markers, and classified the cohort into three distinct subgroups with clear separation, termed Classical pattern (N = 85), Transitional pattern (N = 85) and Basal-like pattern (N = 20), respectively (Fig. 3a). We evaluated five clustering methods (Ward, Hybrid Ward, Average, Centroid, and Complete) using JMP 17.0.1. (Supplementary Fig. 3). The dendrograms indicated that the Ward and Hybrid Ward methods effectively differentiated the classifications, with both methods yielding identical clustering outcomes. Because of this, the Ward method was adopted for further analysis. Classical pattern tended to show high expression (score 2+ or 3+) of HNF4a and GATA6 with low expression (score 0 or 1+) of CK5/6 and/or p63, whereas Basal-like pattern generally demonstrated the opposite trend (Fig. 3b). These IHC-based three clusters were significantly associated with histological categories (*p* < 0.001, Fig. 3b, Table 2), Classical and Basal-like pattern were related to histological Glandular pattern and squamous differentiation, respectively (Fig. 3b and Table 2).

**Table 1:** Expression of the four markers.

	0	1+	2+	3+
CK 5/6	146 (77%)	21 (11%)	9 (5%)	14 (7%)
p63	125 (66%)	43 (22%)	14 (7%)	8 (4%)
GATA6	2 (1%)	13(7%)	60 (31%)	115 (61%)
HNF4a	28 (15%)	31 (16%)	65 (34%)	66 (35%)

**Table 2:** Clinicopathologic characteristics of the marker based groups.

				Total			Classical			Transitional		Basal-like		*p* value	
Stage		I			15			8			7		0		0.476
		II			23			13			8		2		
		III			37			17			14		6		
		IV			107			42			53		12		
Sex			Male			96			44	45		7		0.359	
			Female			94			41	40		13			
Age	< 70			86			38			42		6		0.314	
	> = 70			104			47			43		14			
Histology	Glandular pattern			134			61			73		0		** < 0.001**	
	w. Por component			40			24			12		4			
	w. Sq differentiation			16			0			0		16			

### Prediction of IHC expression status based on histology and IHC pattern

The results showed that “CK5/6 IHC score 3+” or “CK 5/6 IHC score 2+ and p63 IHC score 2+” or “CK5/6 IHC score 2+ and Sq diff histology” is efficiently classified as IHC basal-like pattern with sensitivity 95.0% and specificity 100% (Supplementary Table 2). Although “CK5/6 IHC score 2+ and Por comp histology” provides an equivocal result for classification, simultaneous GATA6 and HNF4a IHC results contribute to more accurate classification because “GATA6 IHC score 3+ and HNF4a IHC score 3+” predicts IHC classical pattern with sensitivity 59.8%, specificity 100% (Supplementary Table 3). On the contrary, “CK5/6 IHC score 0 or p63 IHC score 0” predicts “not IHC basal-like pattern” with sensitivity 92.9%, specificity 100% (Supplementary Table 4). Thus, the combination of histological evaluation and IHC scoring of four markers seems to provide an approximate prediction of IHC classification and the associated molecular signature.

### Clinical characteristics of IHC expression types

IHC expression pattern was not related to clinical stage or sex and age (Table 2). Kaplan–Meier analysis demonstrated significant worst prognosis of Basal-like pattern patients amongst all three groups (Log-rank *p* = 0.004, Fig. 3c). Cox proportional hazards model showed IHC expression status (Transitional, Basal-like vs Classical) was a significant prognostic variable by both univariate analysis [*p* = 0.002, Hazard ratio (95% CI) 1.89 (1.27–2.83)] and multivariate analysis [*p* = 0.047, Hazard ratio (95% CI) 1.58 (1.01–2.38)]. On the other hand, histological status (with vs without squamous differentiation) was not an independent prognostic variable by multivariate analysis [*p* = 0.067, Hazard ratio (95% CI) 1.77 (0.85–3.26)] (Table 3).

**Table 3:** Prognostic factors of PDAC.

		Univariate analysis		Multivariate analysis	
		HR (95% CI)	*p* value	HR (95% CI)	*p* value
Age	(> = 70 vs < 70)	0.844 (0.578–1.233)	0.381	0.840 (0.554–1.274)	0.413
Sex	(male vs female)	1.133 (0.776–1.654)	0.669	1.119 (0.748–1.673)	0.516
Stage	(III, IV vs I , II)	1.916 (1.156–3.174)	**0.012**	1.913 (1.130–3.240)	**0.019**
Histology	(with vs without squamous differentiation)	2.236 (1.292–3.868)	**0.004**	1.771 (0.851–3.262)	0.067
Expression type	(transitional, basal-like vs classical)	1.893 (1.268–2.825)	**0.002**	1.578 (1.006–2.380)	**0.047**

### CK5/6 positivity in histological Glandular pattern PDAC

**Figure 4:**
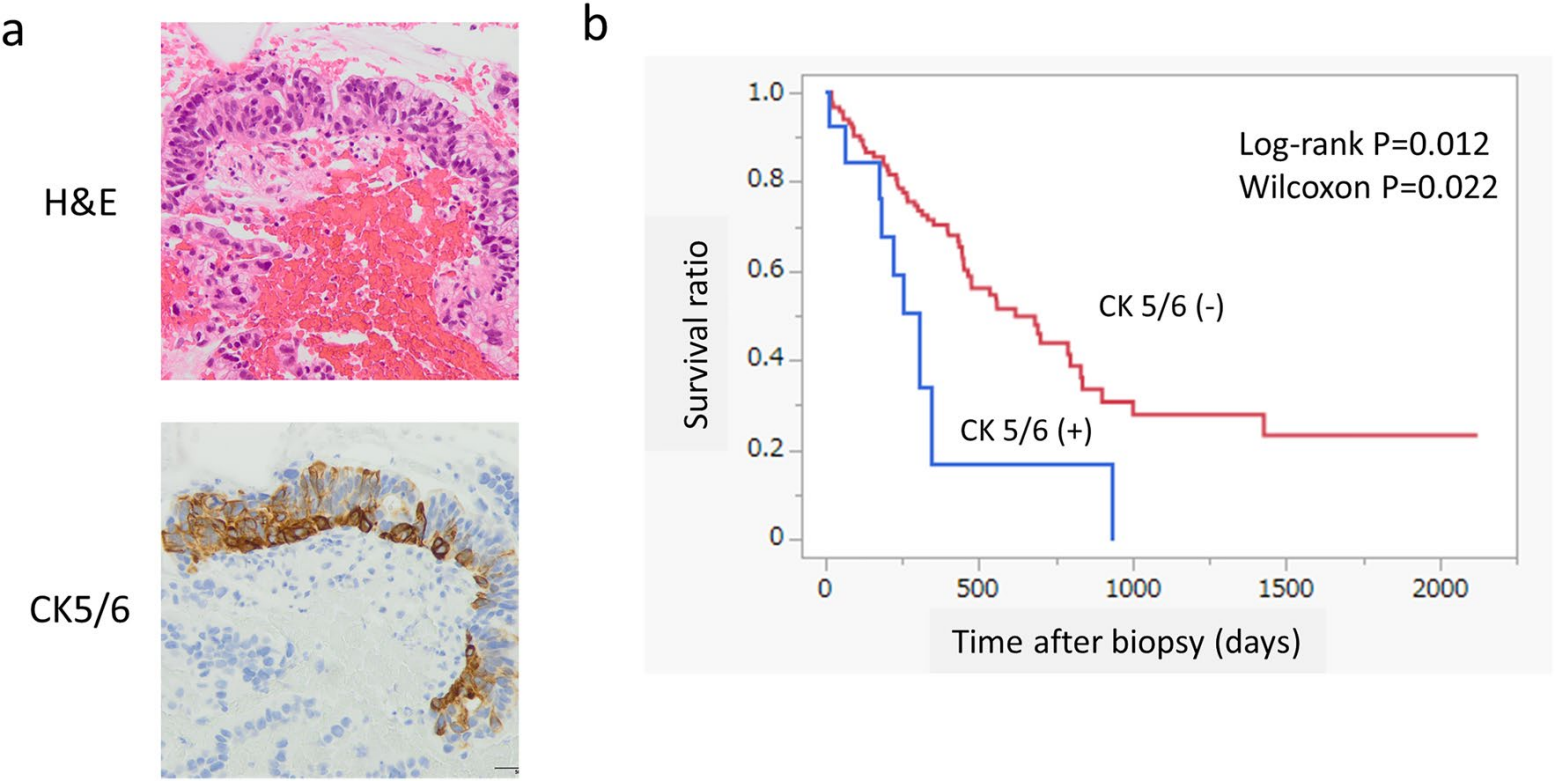
Clinical significance of CK5/6 positivity in histological glandular PDAC. (**a**) Glandular pattern PDAC cells focally express CK5/6. (**b**) Within glandular pattern PDAC, CK5/6 positive subgroup showed significantly poorer prognosis than CK5/6 negative subgroup (Log-rank *p* = 0.012, Wilcoxon *p* = 0.022).

Within the histological Glandular pattern cases (N = 134), CK5/6 positive PDAC (N = 14) (Fig. 4a) showed significantly poorer prognosis compared to CK5/6 negative PDAC (N = 120) (Log-rank *p* = 0.012, Fig. 4b). CK5/6 positive status (score 1+ , 2+, 3+ vs score 0) was an independent prognostic factor by both univariate analysis [*p* = 0.015, Hazard ratio (95% CI) 2.44 (1.19–5.00)] and multivariate analysis [*p* = 0.008, Hazard ratio (95% CI) 2.82 (1.31–6.08)] among histological Glandular population (Supplementary Table 5. Nevertheless, p63 positive status revealed no significant association with prognosis in this situation on both univariate and multivariate analysis [*p* = 0.714, Hazard ratio (95% CI) 1.13 (0.58–2.91)] (Supplementary Table 5).

## Discussion

In this study, we showed the usefulness of the representative PDAC subtyping markers (TP63, KRT5, KRT6A, HNF4a and GATA6) and its clinicopathologic meanings with treatment naïve consecutive EUS-FNAB samples. A recent study showed that CK5 and/or GATA6, along with software analysis of digital images, are useful markers for expressional subtyping^15^. However, we believe that pathologists can perform subtyping based on their daily IHC evaluation if our combination is adopted.

Based on several prior data as to molecular mechanism for the acquisition of basal phenotype with loss of classical gene program in PDAC progression^9–13,16,17,21,22^ and gene expression profile from our re-evaluation of TCGA data sets, we selected four representative IHC candidates (GATA6, HNF4a, CK5/6, p63) for predicting molecular signatures. IHC expression subtypes (Classical, Transitional, Basal-like) based on IHC scores for these markers demonstrated a significant association with the histological subtypes and prognosis; IHC basal-like status is significantly related to histological squamous differentiation and poorer prognosis compared to IHC classical subtype. Since the reference transcriptomic subclassification data for each case was not established through RNA-seq in this study, it is controversial whether this IHC expression classifier is directly associated with molecular subtypes. Nevertheless, given that clinicopathological characteristics of IHC basal-like status overlap with those of molecular basal-like signature described in previous studies^12,23^, it suggests that IHC expression classifier is a potential surrogate biomarker for detection of molecular basal-like subgroup which is of clinical importance. Previous clinical and preclinical findings demonstrated that basal-like PDAC was less sensitive to standard chemotherapy, including mFOLFIRINOX or gemcitabine-based regimens, than the classical subtype^10,11,24^. Additionally, a multi-institutional randomized phase II trial to evaluate molecular subtypes as biomarkers for therapy response is in progress^25^, indicating that IHC subtyping has the potential to contribute to prognostic stratification and personalized treatment selection.

Although recent studies demonstrated that GATA6 expression by RNA-seq, ISH and IHC with digital assistance distinguished transcriptomic classical and basal-like subtype in advanced PDAC^10,13^, it is difficult to implement these technologies into clinical practice because it is costly, time-consuming and is limited to access only in high-volume cancer centers. In this regard, our methodology successfully overcomes these three limitations due to applying only four commercially available IHC markers to daily diagnostic practice. As previous studies have noted^13^, subjective evaluation of GATA6 staining intensity by pathologists is less accurate. The combination of GATA6 and three additional IHC markers, however, is expected to improve the interpretation discrepancy between pathologists for the reasons described in results.

Recent studies including single-cell resolutions have indicated complex spatial and temporal intratumor heterogeneity of PDAC^11,12^. Both classical and basal-like cells co-exist within a single PDAC patient in various proportions, and basal-like expression program is acquired during disease progression. Furthermore, it has been revealed that recurrent PDAC harbored an altered genomic landscape through treatment-induced genetic bottleneck^26^. As the present analysis focuses on biopsied materials taken from primary lesions of treatment-naïve patients, it is possible that basal-like cells hidden in primary lesions outside the biopsy site and/or metastatic lesions are underestimated, and it is difficult to evaluate basal-like subclones developed in recurrent lesions after treatment. Indeed, the fact that patients exhibiting focal CK5/6 expression despite glandular histology (not classified as IHC basal-like pattern) are associated with poor prognosis may indicate that intratumor heterogeneity with basal-like subclones stemming from classical clones is critical for PDAC outcome^12^. Further validation analyses using matched EUS-FNAB samples with gene expression profiles obtained from liquid biopsy specimens that provides spatial comprehensive genetic landscape are needed to evaluate how accurately this IHC expression classifier model reflects the impact of basal-like subclones behind the biopsy site. In addition, expanding the cohort to post-treatment patients is leveraged to estimate whether a similar model is applicable to PDAC post-neoadjuvant chemotherapy.

In addition to the challenges facing the IHC classifier model mentioned above, this study has several limitations. First, we excluded cases in which the FNAB material was insufficient for pathological diagnosis and additional IHC assessment. Whether this IHC classifier applies to FNAB samples with only a small number of atypical tumor cells remains unknown. Second, the treatment information for this cohort and possible factors affecting patient prognosis, including treatment type (surgery and/or chemotherapy) and chemotherapy regimens, were not considered in the multivariate analysis. Further validation studies on this cohort, including cases where FNAB samples have few atypical cells and/or treatment factor-adjusted analyses, are needed.

In conclusion, our study shows that IHC expression classification based on combined four IHC markers (CK5/6, p63, GATA6, HNF4a) is a versatile surrogate biomarker predicting transcriptomic subgroup in treatment-naïve advanced PDAC. Although further validation analyses are needed to be applied to personalized medicine for PDAC, our results propose the basis for the utility of a prognostic stratification model using histopathological and immunohistochemical evaluation of EUS-FNAB samples.

### Supplementary Information

Supplementary Information.

## Data Availability

The datasets used and/or analyzed during this study are available from the corresponding author on reasonable request.
