# Supplementary material for: Combination immunohistochemistry for CK5/6, p63, GATA6, and HNF4a predicts clinical outcome in treatment-naïve pancreatic ductal adenocarcinoma
Source: Sci Rep. 2024 Jul 6;14:15598. doi: 10.1038/s41598-024-65900-w (PMC11227498; doi:10.1038/s41598-024-65900-w)
Supplement: Supplementary file 1 — Supplementary Information. [file 41598_2024_65900_MOESM1_ESM.docx]

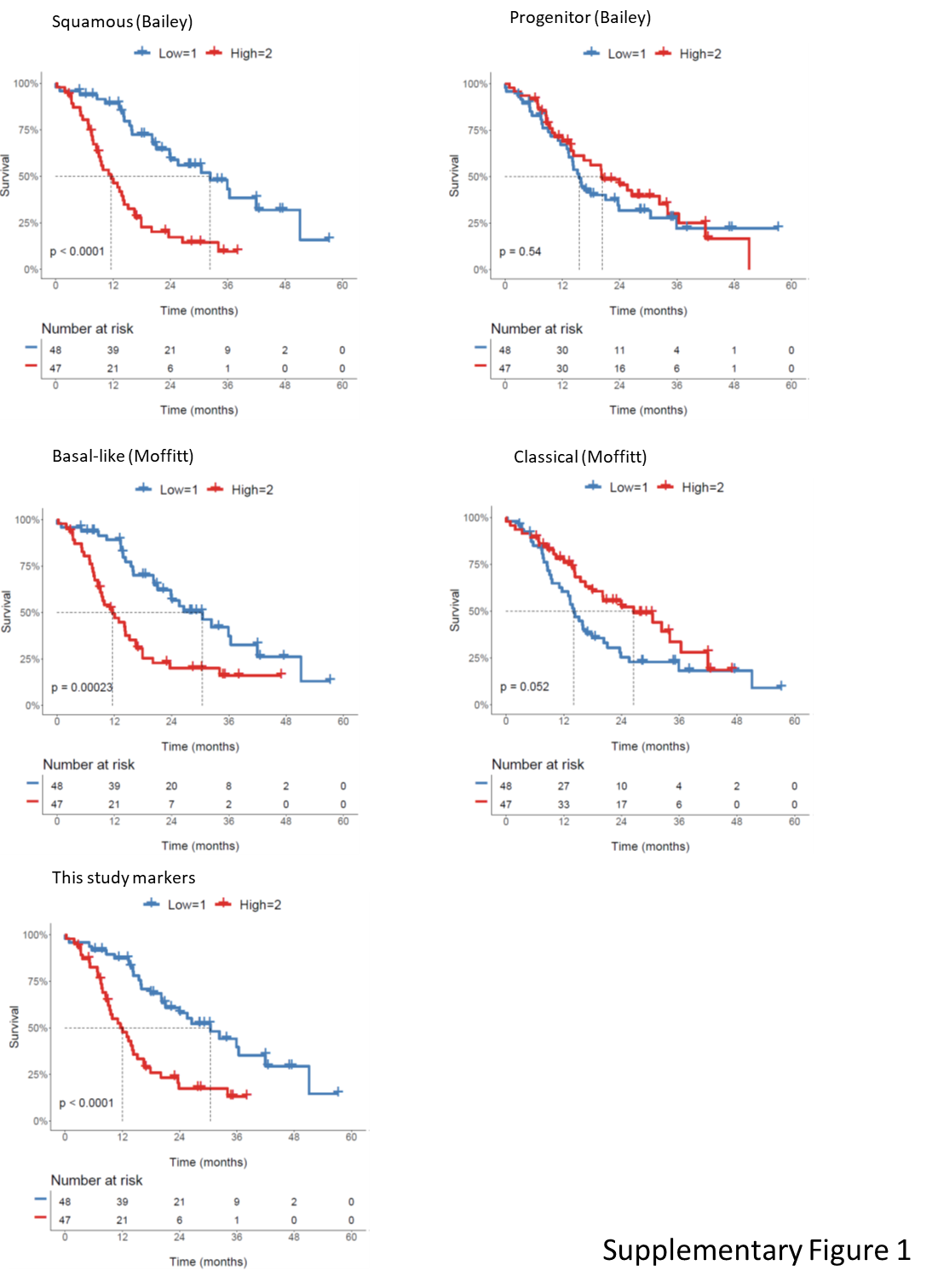


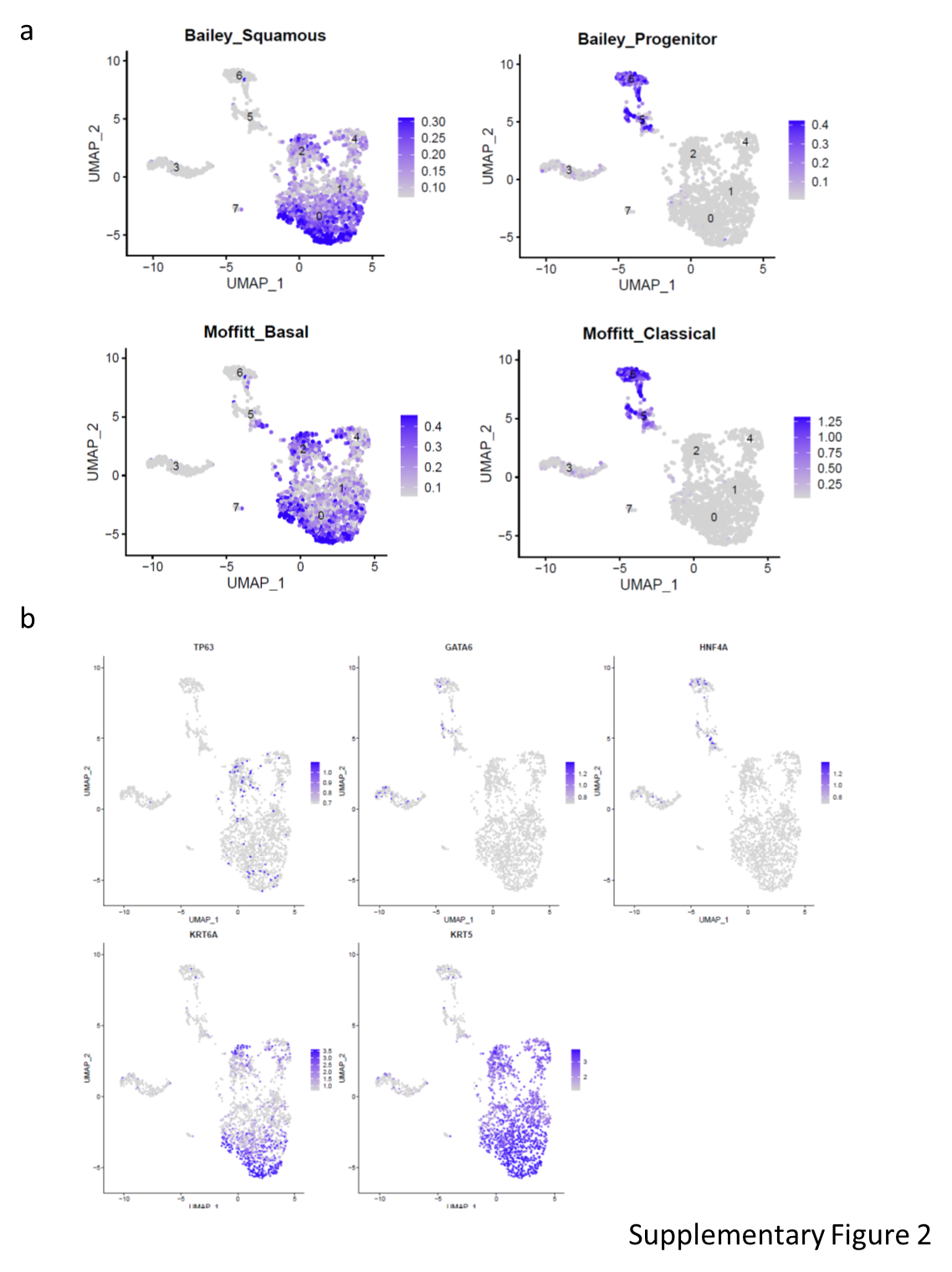


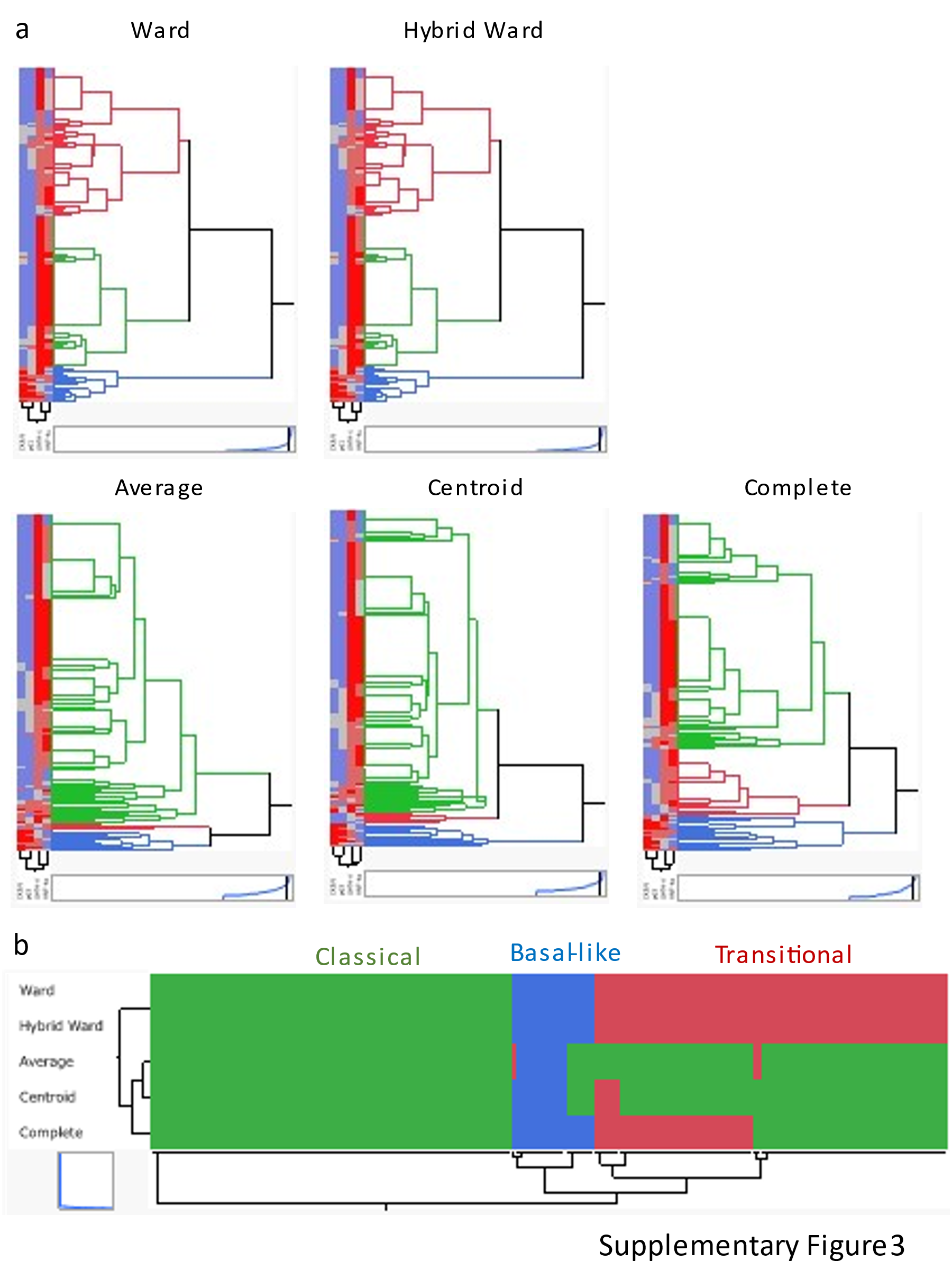


**Supplementary Figure 1.**Prognosis in the Squamous/Basal-like and Squamous/Progenitor subtypes and Markers used in this study. Among the five markers, TP63, KRT5, and KRT6A were used as positive markers, while GATA6 and HNF4a were used as negative markers. Differences between positive markers and negative markers were used to dichotomize patients.

**Supplementary Figure 2.**UMAP plot of gene signature scores based on single-cell RNA-seq data for patient 85948 from Chan-Seng-Yue et al. (2020) using (a) subtype markers defining Squamous, Progenitor, Classical, and Basal-like subtypes (Bailey and Moffitt) and (b) our five markers.

**Supplementary Figure 3.** The dendrograms of five unsupervised hierarchical clustering methods, Ward, Hybrid Ward, Average, Centroid, and Complete (a), indicated that the Ward and Hybrid Ward methods effectively differentiated the classifications with identical clustering outcomes (b).

**Supplementary Table 1. Clinicopathologic Characteristics of This Study Cohort**

| Clinicopathologic Factors | | Cases |
| --- | --- | --- |
| Stage | IA | 9 |
|  | IB | 6 |
|  | IIA | 16 |
|  | IIB | 7 |
|  | III | 37 |
|  | IV | 107 |
|  |  |  |
| Sex | Male | 96 |
|  | Female | 94 |
|  |  |  |
| Age | < 70 | 86 |
|  | >= 70 | 104 |
|  |  |  |
| Histology | Glandular pattern | 134 |
|  | with Poorly differentiated component | 40 |
|  | with Squamous differentiation | 16 |

**Supplementary Table 2. Prediction of IHC expression status based on histology and IHC pattern**

**Supplementary Table 3. Prediction of IHC expression status based on histology and IHC pattern**

**Supplementary Table 4. Prediction of IHC expression status based on histology and IHC pattern**

**Supplementary Table 5. Prognostic Factors of Glandular Type PDAC**

|  |  | **Univariate analysis** | |  | **Multivariate analysis** | |
| --- | --- | --- | --- | --- | --- | --- |
|  |  | HR (95%CI) | P value |  | HR (95%CI) | P value |
| **Age** | ( >=70 vs <70) | 0.836 (0.509-1.375) | 0.481 |  | 0.995 (0.583-1.700) | 0.987 |
| **Sex** | (male vs female) | 1.031 (0.633-1.681) | 0.901 |  | 1.145 (0.664-1.974) | 0.627 |
| **Stage** | (III, IV vs I , II) | 1.996 (1.071-3.720) | **0.030** |  | 2.383 (1.215-4.672) | **0.012** |
| **CK 5/6** | (score 1,2,3 vs score 0) | 2.436 (1.187-4.998) | **0.015** |  | 2.822 (1.311-6.079) | **0.008** |
| **p63** | (score 1,2,3 vs score 0) | 1.086 (0.616-1.914) | 0.775 |  | 1.132 (0.584-2.914) | 0.714 |
| **GATA6** | (score 0,1 vs score 2,3) | 1.233 (0.170-8.966) | 0.836 |  | 1.285 (0.171-9.649) | 0.808 |
| **HNF4a** | (score 0,1 vs score 2,3) | 1.268 (0.742-2.167) | 0.385 |  | 1.401 (0.794-2.490) | 0.242 |
